# Psychological distress, digital behavioral risks, and physical activity as predictors of subjective wellbeing: a machine learning study in a large sample of community-dwelling adults

**DOI:** 10.3389/fnbeh.2026.1876257

**Published:** 2026-07-08

**Authors:** Mouna Saidane, Noomen Guelmami, Wissem Dhahbi, Fida Ayachi, Halil İbrahim Ceylan, Lolwa Barakat, Noureddine M. Ben Said, Mohammed Issa Alsaeed, Raul Ioan Muntean, Ismail Dergaa

**Affiliations:** 1 Department of Human and Social Sciences, High Institute of Sport and Physical Education of Kef, University of Jendouba, Kef, Tunisia; 2 Sport Sciences, Health and Movement, High Institute of Sport and Physical Education of Kef, University of Jendouba, Kef, Tunisia; 3 Training Department, Police College, Police Academy, Doha, Qatar; 4 Physical Education of Sports Teaching Department, Faculty of Sports Sciences, Atatürk University, Erzurum, Türkiye; 5 Clinical Research Center, USC Institute for Addiction Science, Keck School of Medicine, University of Southern California, Los Angeles, CA, United States; 6 Department of Biomechanics and Motor Behavior, College of Sport Sciences and Physical Activity, King Saud University, Riyadh, Saudi Arabia; 7 Department of Physical Education and Sport, Faculty of Law and Social Sciences, University “1 Decembrie 1918” of Alba Iulia, Alba Iulia, Romania; 8 High Institute of Sport and Physical Education of Ksar-Said, University of Manouba, Manouba, Tunisia

**Keywords:** compulsive internet use, machine learning, nomophobia, physical activity, psychological distress, subjective wellbeing

## Abstract

**Background:**

Regular physical activity is a key determinant of psychological wellbeing, yet its interaction with emerging digital behavioral risks remains insufficiently understood. Compulsive internet use and nomophobia have been linked to psychological distress, which negatively affects life satisfaction and happiness. However, few studies have examined these factors simultaneously. Machine learning offers a promising approach for improving predictive accuracy and clarifying the relative contributions of these variables to subjective wellbeing.

**Aim:**

This study examined the combined predictive roles of physical activity, psychological distress, compulsive internet use, and nomophobia in subjective wellbeing (life satisfaction and happiness) and identified the optimal machine learning models for each outcome.

**Methods:**

A cross-sectional study was conducted among 1,479 community-dwelling adults (51.05% males) recruited between September and December 2024. Participants completed validated Arabic versions of the IPAQ-SF, DASS-21, CIUS, NMP-Q, SWLS, and SHS. Six machine learning algorithms (linear regression, random forests, support vector machines, XGBoost, k-nearest neighbors, and LASSO) were trained using an 80/20 train–test split with 5-fold cross-validation. Performance was evaluated using *R*^2^, RMSE, and MAE.

**Results:**

Psychological distress was the strongest negative predictor of wellbeing, showing the largest associations with happiness (*r* = −0.40) and life satisfaction (*r* = −0.37). Compulsive internet use was moderately associated with distress (*r* = 0.48), whereas nomophobia showed negligible relationships with both outcomes. For subjective happiness, random forest achieved the best performance (*R*^2^ = 0.154, RMSE = 0.869), slightly outperforming linear regression and LASSO (*R*^2^ = 0.153). For life satisfaction, linear regression and LASSO performed best (*R*^2^ = 0.129, RMSE = 1.317), while support vector machines showed the lowest accuracy. Higher physical activity levels, particularly vigorous activity, were consistently associated with more favorable psychological profiles.

**Conclusion:**

Prediction of subjective wellbeing is outcome-specific, with different machine learning models performing optimally for happiness and life satisfaction. Psychological distress emerged as the strongest negative predictor, whereas physical activity demonstrated a consistent protective association. Longitudinal studies are needed to clarify causal pathways linking digital behavioral risks to wellbeing.

## Graphical abstract

**Figure:**
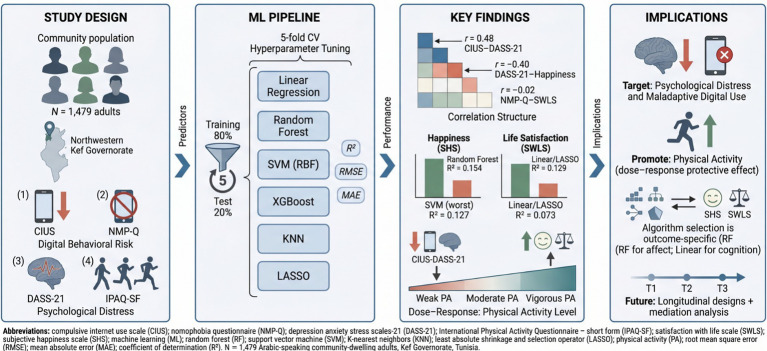

## 1 Introduction

The global proliferation of internet-connected devices has fundamentally altered patterns of human behavior, social interaction, and mental health. Internet adoption in North African and Arab-speaking regions accelerated substantially following the COVID-19 pandemic, intensifying both the frequency and the compulsive quality of digital engagement across community populations. While connectivity has conferred measurable informational and social benefits, a substantial body of evidence indicates that dysregulated patterns of internet use carry meaningful psychological costs (Dhahbi and Ben Saad, 2024; Masaeli and Billieux, 2022). Excessive and unhealthy digital engagement is now recognized as a multidimensional construct encompassing compulsive internet use, nomophobia, and broader manifestations such as digital obesity, defined as the chronic overloading of the cognitive and behavioral system through disproportionate digital consumption (Özbay et al., 2025). Özbay et al. (2025) recently developed and psychometrically validated a Digital Obesity Scale, providing an operational framework for assessing this construct in community populations. Concurrently, the emergence of smartphone-specific behavioral constructs, most notably nomophobia (fear of mobile phone unavailability), has introduced dimensions of digital-behavioral risk that remain imperfectly characterized outside Western clinical samples. Subjective wellbeing, operationalized through its cognitive-evaluative component (life satisfaction) and its affective-experiential component (subjective happiness), represents a fundamental public health outcome for which upstream behavioral determinants are increasingly recognized as modifiable (Diener et al., 2018). Identifying those determinants with sufficient predictive precision, particularly in Arabic-speaking populations, constitutes a pressing scientific priority.

Compulsive internet use (CIU) is characterized by loss of control over online behavior, withdrawal-like symptoms upon disconnection, and the progressive displacement of social and occupational obligations by internet-related activity (Meerkerk et al., 2009). A systematic review by Masaeli and Billieux (2022) documented consistent negative associations between problematic internet and smartphone use and global life satisfaction, while noting that the existing evidence predominantly derives from student samples with limited external validity. More recent cross-sectional evidence in adult community populations indicated a significant inverse association between internet addiction and psychological wellbeing (*r* = −0.417, *p* < 0.001), with excessive online engagement exacerbating symptoms of anxiety, depression, and social disengagement (Arayici et al., 2025). The interconnection between CIU and formal psychological distress, as assessed by the Depression Anxiety Stress Scales (DASS-21), represents a particularly salient pathway, given the likely bidirectionality between dysregulated use and symptom severity. Despite this theoretical plausibility, the relative magnitude of these associations, when examined alongside competing predictors in multivariate or machine-learning frameworks, has been scarcely quantified in Arabic-speaking community samples.

Nomophobia, operationalized across four dimensions (inability to communicate, loss of connectedness, inability to access information, and loss of convenience), has emerged as a recognized behavioral health construct with rapidly growing empirical representation (Yildirim and Correia, 2015). A large-scale cross-sectional study conducted across five Arab countries reported substantial nomophobia prevalence among 5,720 university students, showing that smartphone dependency is a significant public health concern across the Arab region (Naser et al., 2023). A population-based study in Saudi Arabia and Jordan further reported nomophobia prevalence of 51.2%, with prior anxiety history and younger age as significant predictors (Alwafi et al., 2022). A meta-analysis that included collaboration from Tunisian researchers found pooled correlations of nomophobia with anxiety (*r* = 0.31, 95% CI: 0.25–0.38) and insomnia (*r* = 0.56, 95% CI: 0.38–0.75) (Daraj et al., 2023). Whether nomophobia independently predicts reduced life satisfaction and happiness, beyond the variance attributable to CIU and psychological distress, remains empirically unresolved.

Physical activity is recognized as a transdiagnostic protective factor for mental health, operating through both neurobiological mechanisms (endorphin release, HPA axis modulation, neuroplasticity) and psychosocial pathways (self-efficacy, behavioral engagement) (Mahindru et al., 2023). A systematic review and best-evidence synthesis showed that physical activity benefits mental health through multiple independent mediators and is inversely associated with psychological distress across diverse populations (Bouzouraa et al., 2025; White et al., 2024). Despite this established evidence base, the International Physical Activity Questionnaire (IPAQ) is frequently treated as a continuous variable in psychological research, potentially obscuring the qualitatively distinct profiles associated with weak, moderate, and vigorous activity classifications. The extent to which IPAQ-defined activity level moderates the relationship between digital behavioral risk factors and wellbeing in Arabic-speaking community samples remains largely unexplored.

Machine learning algorithms offer methodological advantages in this context: they accommodate non-linear interaction effects without explicit specification, make fewer parametric assumptions, and generate variable importance metrics that quantify each predictor’s relative contribution (Dhahbi and Chamari, 2026; Fife and D'Onofrio, 2023). Across a multi-site study of 17 universities in Southeast Asia, random forests and adaptive boosting achieved the highest accuracy for identifying negative mental wellbeing traits, with physical activity among the top five most salient predictive features. Despite this progress, systematic multi-algorithm comparisons using comprehensive digital-behavioral predictor sets remain scarce in Arabic-speaking contexts, and no study has simultaneously compared algorithm performance across the conceptually distinct outcomes of life satisfaction and subjective happiness.

Despite this body of evidence, three specific gaps justify the present study. First, no published study has simultaneously modeled CIU, nomophobia, psychological distress, and physical activity as predictors of two distinct wellbeing outcomes within a unified ML framework in an Arabic-speaking community sample; existing work addresses these constructs in isolation or in Western or Asian student populations with limited generalizability (Masaeli and Billieux, 2022; Naser et al., 2023). Second, prior ML applications to wellbeing prediction have either used a single algorithm or compared algorithms on a single outcome, precluding systematic evidence on outcome-specific algorithm selection (Kim et al., 2023). Third, the distributional relationship between IPAQ-classified physical activity levels and digital behavioral risk constructs has not been characterized simultaneously with wellbeing outcomes, leaving the dose–response structure of this relationship empirically undocumented in the target population (Bouzouraa et al., 2025; White et al., 2024).

The present study makes three specific contributions to the literature. Conceptually, it is among the first to examine CIU, nomophobia, and DASS-21-defined psychological distress alongside IPAQ-SF-classified physical activity within a single predictive framework in an Arabic-speaking community sample, a population that is underrepresented in behavioral and digital health research. Methodologically, it applies a multi-algorithm ML comparison across two conceptually distinct wellbeing outcomes within the same dataset, enabling outcome-specific algorithm selection rather than assuming a universal best-performing model. Analytically, it characterizes the distributional gradient of all five study variables simultaneously as a function of IPAQ-defined activity level, providing evidence for a monotonic dose–response relationship across both risk and protective constructs.

The present study addresses these gaps through three pre-specified research questions. RQ1: What are the bivariate associations among CIU (CIUS), psychological distress (DASS-21), physical activity (IPAQ-SF), and nomophobia (NMP-Q) with life satisfaction (SWLS) and subjective happiness (SHS) in an Arabic-speaking community sample (*N* = 1,479)? Which ML algorithm achieves optimal predictive performance for each of the two wellbeing outcomes when evaluated on a held-out test set using RMSE, MAE, and *R*^2^? How are the five study variables distributed across IPAQ-defined physical activity levels, and is there evidence of a monotonic dose–response gradient? Applying a rigorous machine learning pipeline to validated Arabic-language instruments, the study provides evidence-based insights for clinicians and policymakers seeking to identify modifiable determinants of wellbeing in Arabic-speaking populations.

The remainder of the manuscript is organized as follows. Section 2 describes the study design, participants, instruments, and statistical analysis pipeline. Section 3 reports the bivariate correlations, ML algorithm performance metrics, and physical activity gradient findings. Section 4 discusses the principal findings in relation to the existing literature, addresses the study’s limitations, and formulates practical recommendations. Section 5 presents the conclusions.

## 2 Methods

### 2.1 Study design and setting

This study employed a cross-sectional observational design to examine the predictive relationships between compulsive internet use, psychological distress, physical activity level, nomophobia, and two distinct wellbeing outcomes (life satisfaction and subjective happiness) in an Arabic-speaking community sample. Cross-sectional designs are appropriate for predictive modeling with validated psychometric instruments when directional causal inference is not the primary objective (Levin, 2006). Data collection occurred between September and December 2024 across multiple sites in the Kef Governorate of northwestern Tunisia, encompassing urban districts of El Kef city and surrounding rural delegations. This geographic stratification ensured adequate demographic diversity within the target population.

### 2.2 Participants

A community-based sample of 1,479 adults (males: *n* = 755, 51.05%; females: *n* = 724, 48.95%) was recruited through convenience and snowball sampling across public universities, secondary schools, primary healthcare centers, community centers, and high-footfall public spaces. Marital status comprised married (48.41%), single (41.51%), and other categories (divorced, widowed, or separated: 10.07%). Educational attainment was nearly equally distributed (below baccalaureate: 51.25%; above baccalaureate: 48.75%), and urban and rural residents constituted 60.99 and 39.01% of the sample, respectively. Inclusion criteria required participants to be aged 18 years or older, to be residents of the Kef Governorate for at least 12 consecutive months, to be Arabic-fluent, and to be able to provide written informed consent. Exclusion criteria comprised severe psychiatric disorders requiring hospitalization, cognitive impairments precluding questionnaire comprehension, and incomplete responses on any scale. The achieved sample size substantially exceeded the minimum required to detect a small-to-medium effect (*f*^2^ = 0.06) with four predictors at alpha = 0.05 and powe*r* = 0.80 [*N*_min = 177; (Faul et al., 2009)], ensuring high statistical power for both correlational and machine learning analyses. Demographic characteristics are presented in Table 1.

**Table 1:** Sociodemographic characteristics of the study sample (*N* = 1,479).

Variable	Category	*n*	%
Sex	Male	755	51.05
	Female	724	48.95
Marital status	Single	614	41.51
	Married	716	48.41
	Other (divorced, widowed, or separated)	149	10.07
Educational attainment	Below baccalaureate	758	51.25
	Above baccalaureate	721	48.75
Residential setting	Urban	902	60.99
	Rural	577	39.01

“Other” marital status encompasses divorced, widowed, and separated participants. Educational attainment was dichotomized at the baccalaureate threshold, consistent with the Tunisian national qualification framework. Urban and rural classifications follow the administrative delimitations of the Kef Governorate, Tunisia.

### 2.3 Procedures

Data collection was conducted by trained research assistants deployed across all recruitment sites, each of whom delivered standardized verbal explanations of study objectives, procedures, potential risks, and confidentiality guarantees before obtaining written informed consent from all participants. For participants with limited literacy, questionnaire items were read aloud, and responses were recorded by the assistant to ensure full comprehension and accurate responses. Questionnaire completion required approximately 20 to 25 min. No personal identifiers were collected at any stage; each completed questionnaire was assigned a unique numeric code, and all data were stored in an encrypted, password-protected database accessible solely to the principal investigator, in accordance with established data security standards (Harriss et al., 2017). Participation was entirely voluntary, and participants retained the explicit right to withdraw at any time without consequence. The research protocol received full ethical approval from the Institutional Review Board of the Higher Institute of Sport and Physical Education of El Kef, University of Jendouba, Tunisia (approval code: ISSEPK0033/2025; December 20, 2025), and all procedures conformed to the principles of the Declaration of Helsinki governing human research (World Medical Association, 2013), Upon completion of the questionnaire, all participants received a psychoeducational pamphlet covering evidence-based guidance on healthy internet use, stress management strategies, and physical activity recommendations.

### 2.4 Measures

All six instruments were administered in their validated Arabic versions in a fixed order to minimize sequence effects. Table 2 summarizes the psychometric properties of each scale in the present sample.

**Table 2:** Psychometric properties of study instruments.

Instrument	Items	Response scale	Score range	Subscales	Cronbach’s *α* (Arabic Validation)	Structural validity	References
CIUS	14	5-point Likert (0–4)	0–56	Unidimensional	0.85–0.90	Single-factor	Meerkerk et al. (2009)
DASS-21	21	4-point Likert (0–3)	0–42 per subscale	Depression, anxiety, stress (7 items each)	>0.90 (total); 0.80–0.89 (subscales)	Three-factor	Moussa et al. (2017)
IPAQ-SF	7	Frequency/duration (continuous)	Categorical: weak/moderate/vigorous	N/A (categorical output)	N/A	Ordinal classification	Craig et al. (2003)
NMP-Q	20	7-point Likert (1–7)	20–140	Communication, connectedness, information, convenience	>0.92	Four-factor (three-factor in some Arab samples)	Yildirim and Correia (2015)
SWLS	5	7-point Likert (1–7)	5–35	Unidimensional	0.82–0.89	Single-factor	Diener et al. (1985)
SHS	4	7-point Likert (1–7)	4–28	Unidimensional	0.80–0.85	Single-factor	Lyubomirsky and Lepper (1999)

CIUS, Compulsive Internet Use Scale; DASS-21, Depression Anxiety Stress Scales-21; IPAQ-SF, International Physical Activity Questionnaire-Short Form; NMP-Q, Nomophobia Questionnaire; SWLS, Satisfaction With Life Scale; SHS, Subjective Happiness Scale.

All instruments were administered in validated Arabic versions. Cronbach’s alpha values fall within the ranges reported in prior Arabic-language validation studies. IPAQ-SF classification follows standard International Consensus Group scoring protocols (Craig et al., 2003). Subscale scores for DASS-21 are multiplied by two to permit normative comparison with the full 42-item version.

#### 2.4.1 Compulsive internet use scale (CIUS)

The 14-item CIUS (Meerkerk et al., 2009) assesses five dimensions of problematic internet use (loss of control, withdrawal, neglect of obligations, interpersonal conflict, and mood regulation) on a five-point Likert scale (0–4; range: 0–56). Higher scores indicate greater severity. Arabic validation studies report Cronbach’s alpha values of 0.85–0.90 across community and student samples.

#### 2.4.2 Depression anxiety stress scales-21 (DASS-21)

The DASS-21 (Lovibond et al., 1995) comprises 21 items distributed across three seven-item subscales (depression, anxiety, and stress), each rated on a four-point scale (0–3). Raw subscale scores are multiplied by two to align with DASS-42 normative benchmarks (range per subscale: 0–42). Arabic validation has confirmed excellent internal consistency (total scale alpha > 0.90) (Moussa et al., 2017).

#### 2.4.3 International physical activity questionnaire-short form (IPAQ-SF)

The seven-item IPAQ-SF (Craig et al., 2003) captures frequency, duration, and intensity of physical activity over the preceding 7 days across four domains (vigorous activity, moderate activity, walking, and sedentary time). Participants were classified into three ordered levels (weak, moderate, or vigorous) per standard IPAQ scoring protocols. This variable was treated as an ordered categorical predictor in all models.

#### 2.4.4 Nomophobia questionnaire (NMP-Q)

The 20-item NMP-Q (Yildirim and Correia, 2015) measures fear of mobile phone unavailability across four dimensions (inability to communicate, loss of connectedness, inability to access information, and loss of convenience) on a seven-point Likert scale (1–7; range: 20–140). Arabic validation studies consistently report Cronbach’s alpha values exceeding 0.92.

#### 2.4.5 Satisfaction with life scale (SWLS)

The five-item SWLS (Diener et al., 1985) assesses global cognitive evaluations of life satisfaction on a seven-point Likert scale (1–7; range: 5–35). Pan-Arab validation studies report alpha values of 0.82–0.89 with a consistently unidimensional factor structure.

#### 2.4.6 Subjective happiness scale (SHS)

The four-item SHS (Lyubomirsky and Lepper, 1999) measures global subjective happiness on a seven-point Likert scale (range: 4–28). Arabic validation has yielded Cronbach’s alpha values of 0.80–0.85, with convergent validity established through positive correlations with the SWLS and inverse correlations with DASS-21 total scores.

### 2.5 Statistical analysis

All analyses were conducted in Team RC (2024) within the RStudio environment (Dhahbi and Dergaa, 2026). During preprocessing, the IPAQ variable was recoded as an ordered factor (weak < moderate < vigorous) to preserve its ordinal structure. Continuous predictors (CIUS, DASS-21, and NMP-Q) were standardized to z-scores (mean = 0, SD = 1), a prerequisite for distance-sensitive algorithms. Outcome variables (SWLS and SHS total scores) were retained on their original metric scales to maintain clinically interpretable error units. Cases with missing data on any instrument were excluded per the pre-specified exclusion criteria, rendering imputation unnecessary.

Bivariate associations were quantified using Pearson product–moment correlation coefficients (corrplot package), and variable distributions across IPAQ levels were visualized using violin plots combined with boxplots (ggplot2). Principal component analysis (PCA) was conducted to characterize the latent dimensional structure of the predictor set, and results were visualized via a correlation circle plot.

The dataset was partitioned into training (80%) and test (20%) subsets using stratified random sampling with a fixed seed to ensure full reproducibility. Six machine learning algorithms were independently trained for each outcome variable: ordinary least squares multiple linear regression (parametric baseline), random forest (randomForest; Breiman, 2001), support vector regression with a radial basis function kernel (e1071), XGBoost (xgboost; Chen and Guestrin, 2016), K-nearest neighbors regression (FNN), and LASSO regression (glmnet; Friedman et al., 2010). Hyperparameter optimization was performed via five-fold cross-validation on the training set using the caret framework (Kuhn, 2008). Tuned parameters included variables sampled per split and number of trees (random forest: 500 trees fixed), learning rate, maximum tree depth, and subsample ratio (XGBoost), the number of neighbors K tested over odd integers 3–21 (KNN), cost and epsilon (SVM), and the L1 regularization parameter lambda (LASSO, selected at minimum cross-validation error). Model performance on the held-out test set was evaluated using three complementary metrics: RMSE, MAE, and *R*^2^. Variable importance was extracted from random forest models and visualized via lollipop plots scaled by node impurity. Residual distributions were examined graphically to assess homoscedasticity and mean-zero error assumptions across all algorithms.

## 3 Results

### 3.1 Sample characteristics

The final analytical sample comprised 1,479 adults (males: *n* = 755, 51.05%; females: *n* = 724, 48.95%). Married participants constituted the largest subgroup (*n* = 716, 48.41%), followed by single (*n* = 614, 41.51%) and other marital statuses (*n* = 149, 10.07%). Educational attainment was nearly equally distributed between those below (*n* = 758, 51.25%) and above (*n* = 721, 48.75%) the baccalaureate threshold. Urban residents accounted for 60.99% (*n* = 902) of the sample, while rural residents accounted for 39.01% (*n* = 577). Full sociodemographic characteristics are reported in Table 1.

### 3.2 Bivariate associations among study variables

**Figure 1:**
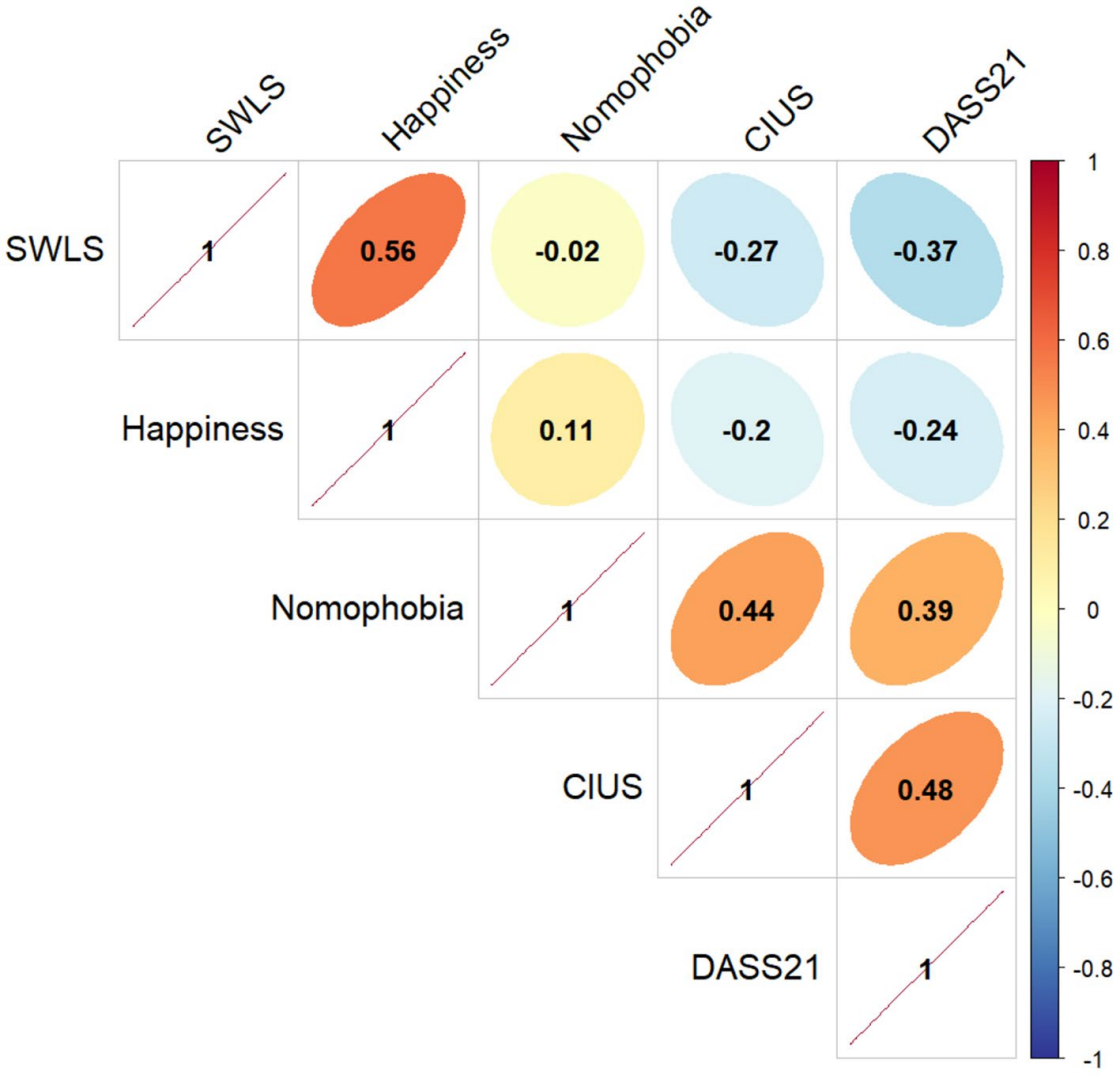
Bivariate Pearson correlation matrix among the five study variables (*N* = 1,479). Cell color intensity reflects the magnitude and direction of each correlation (red = positive; blue = negative). Diagonal cells represent self-correlations (*r* = 1.00). SWLS, Satisfaction with Life Scale; CIUS, Compulsive Internet Use Scale; DASS-21, Depression Anxiety Stress Scales-21.

Pearson correlation coefficients among all five study variables are presented in Table 3 and visualized as a color-coded matrix in Figure 1. The strongest positive association in the matrix was observed between CIUS and DASS-21 (*r* = 0.48), indicating substantial co-occurrence of compulsive internet use and psychological distress. CIUS also correlated positively with nomophobia (*r* = 0.44), and DASS-21 did so as well (*r* = 0.39). These intercorrelations, while statistically significant (*p* < 0.001), indicate that no two predictors shared more than 23% of their variance, supporting adequate discriminant validity among the three constructs.

**Table 3:** Bivariate Pearson correlation matrix among study variables (*N* = 1,479).

Variable	SWLS	Happiness	Nomophobia	CIUS	DASS-21
SWLS	–				
Happiness	**0.56**	–			
Nomophobia	−0.02	0.11	–		
CIUS	−0.27	−0.24	**0.44**	–	
DASS-21	**−0.37**	**−0.40**	0.39	**0.48**	–

SWLS, Satisfaction With Life Scale; CIUS, Compulsive Internet Use Scale; DASS-21, Depression Anxiety Stress Scales-21.

Lower triangle: Pearson r coefficients; upper triangle left blank for clarity. Bold with red cells: *r* > = 0.40 (large positive). Bold with blue cells: *r* < = − 0.35 (large negative).

Regarding wellbeing outcomes, the DASS-21 demonstrated the strongest negative associations with both subjective happiness (*r* = −0.40) and life satisfaction (*r* = −0.37), indicating that psychological distress is the predominant inverse predictor of wellbeing. The slightly stronger association with happiness than with SWLS is theoretically meaningful, as affective distress is more proximal to momentary hedonic experience than to global cognitive life evaluation. CIUS showed moderate negative correlations with SWLS (*r* = −0.27) and happiness (*r* = −0.24). By contrast, nomophobia exhibited negligible associations with both wellbeing outcomes (SWLS: *r* = −0.02; happiness: *r* = 0.11), suggesting no meaningful direct linear relationship between fear of mobile phone unavailability and subjective wellbeing. The two outcome constructs were themselves moderately correlated (*r* = 0.56), suggesting that SWLS and SHS capture related yet empirically distinct dimensions of wellbeing (shared variance = 31.4%), and should be modeled separately.

### 3.3 Comparative machine learning model performance

**Figure 2:**
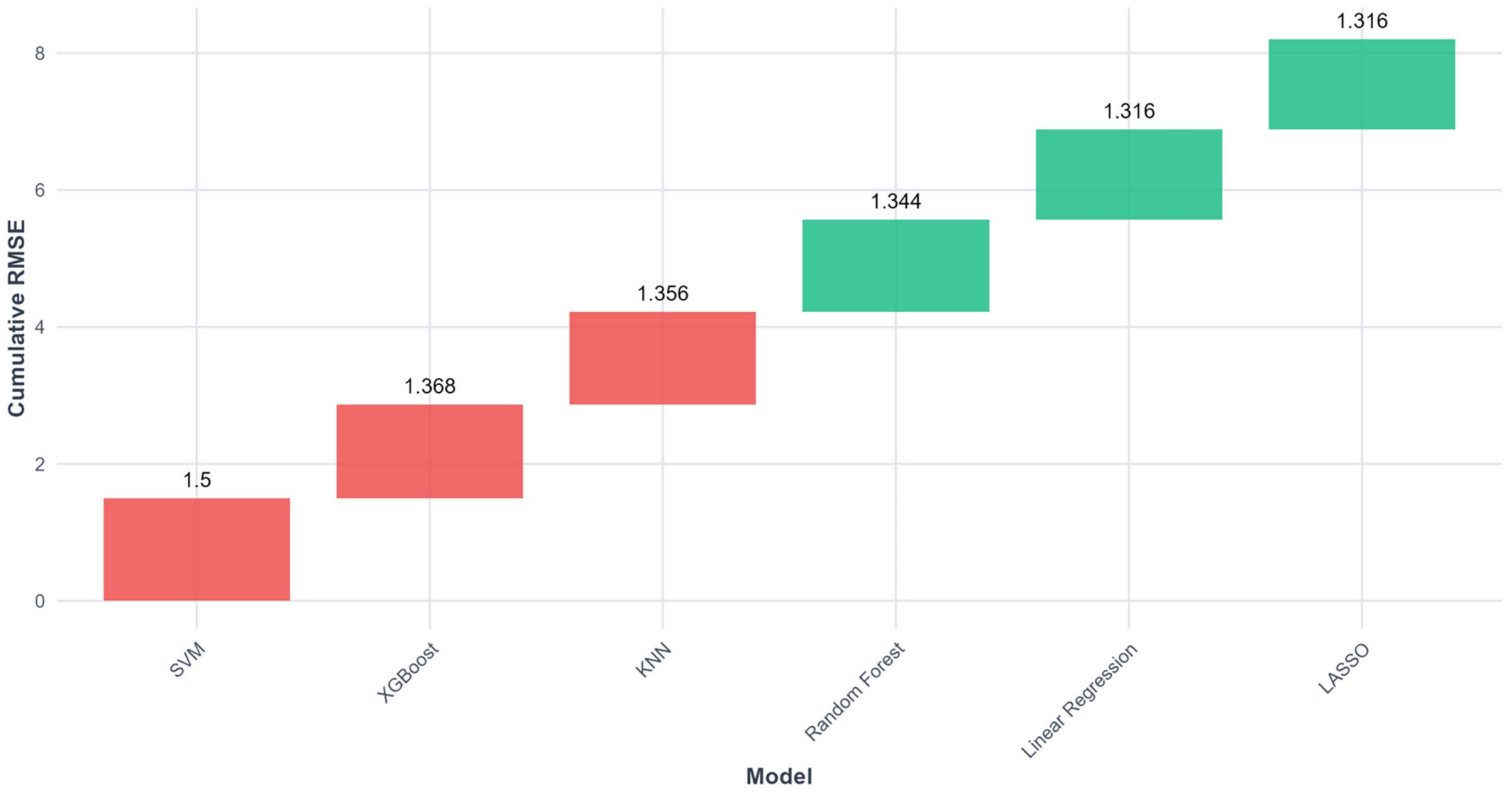
Slopegraph comparing the coefficient of determination (*R*^2^) achieved by each of the six machine learning algorithms for subjective happiness (left axis) and life satisfaction (SWLS; right axis) on the hold-out test set. Lines connect values from the same algorithm across the two outcomes. LASSO, least absolute shrinkage and selection operator; KNN, K-nearest neighbors; SVM, support vector machine.

**Figure 3:**
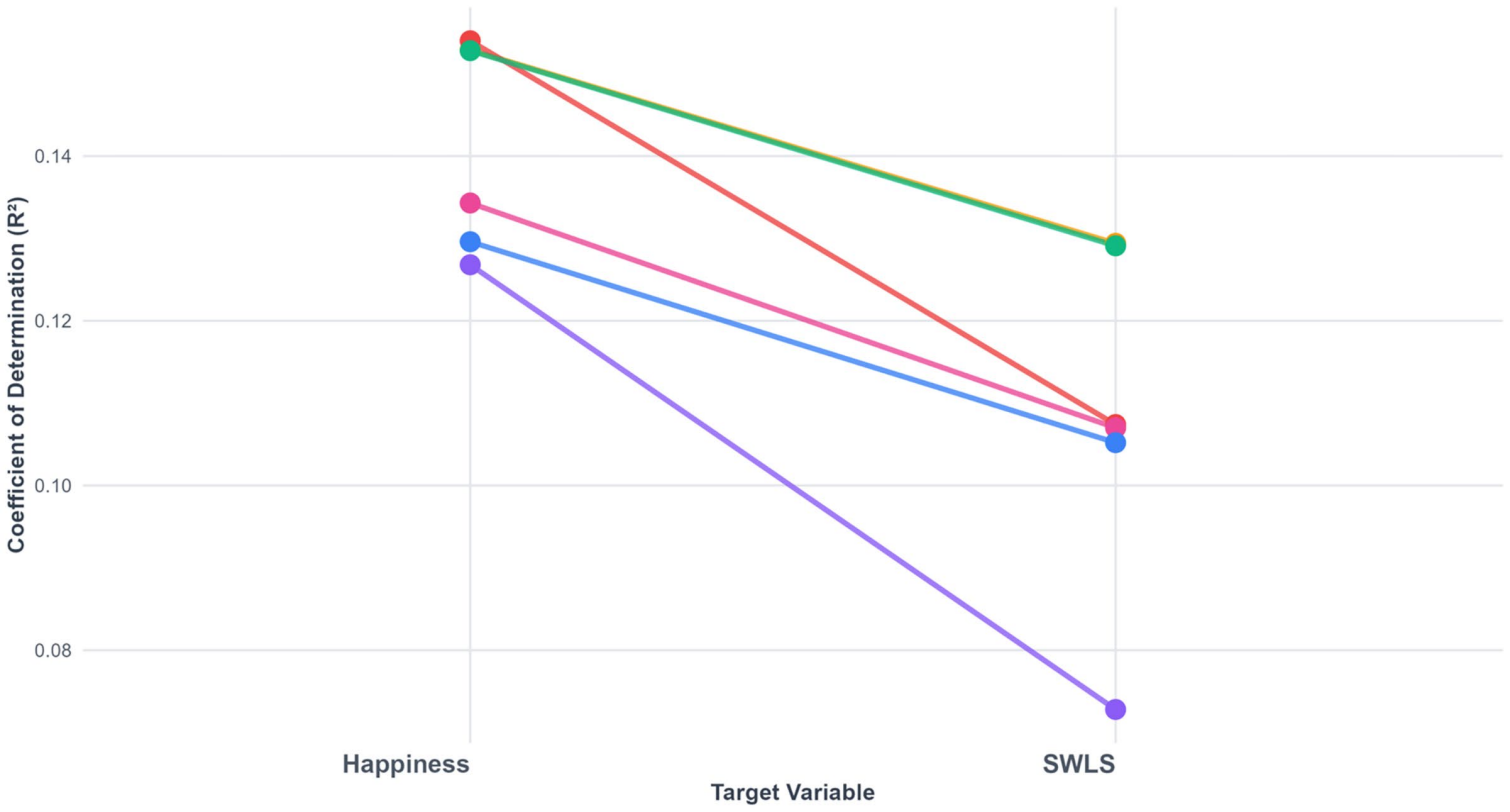
Waterfall chart displaying the individual root mean square error (RMSE) contributed by each of the six machine learning algorithms for life satisfaction (SWLS) prediction on the hold-out test set. Bars are ordered from highest (SVM) to lowest (Linear Regression, LASSO) RMSE. Red bars indicate above-median error; green bars indicate below-median error.

Predictive performance metrics for all six algorithms across both outcomes are presented in Table 4 and compared visually in Figure 2 (slopegraph of *R*^2^ by outcome) and Figure 3 (waterfall chart of cumulative RMSE for SWLS). A consistent pattern emerged: all algorithms produced higher *R*^2^ and lower RMSE values for subjective happiness than for life satisfaction, indicating that the selected predictor set shares a stronger systematic relationship with affective wellbeing than with cognitive-evaluative wellbeing.

**Table 4:** Predictive performance of six machine learning algorithms for subjective happiness and life satisfaction on the hold-out test set (20%; *n* ≈ 296).

Algorithm	Subjective happiness (SHS)			Life satisfaction (SWLS)		
	RMSE	MAE	*R* ^2^	RMSE	MAE	*R* ^2^
Linear regression	0.8690	0.7403	0.1529	**1.3165**	**1.1008**	**0.1294**
Random forest	**0.8687**	0.7361	**0.1540**	1.3444	1.1091	0.1074
SVM (RBF kernel)	0.9482	**0.7092**	0.1268	1.4996	1.1677	0.0728
XGBoost	0.8892	0.7281	0.1343	1.3679	1.1140	0.1070
KNN	0.8865	0.7349	0.1296	1.3558	1.1098	0.1052
LASSO	0.8690	0.7407	0.1528	**1.3165**	1.1011	0.1291

RMSE, root mean square error (original scale units); MAE, mean absolute error; *R*^2^, coefficient of determination. SHS, Subjective Happiness Scale (range 4–28); SWLS, Satisfaction with Life Scale (range 5–35). SVM, support vector machine; RBF, radial basis function kernel; KNN, K-nearest neighbors; LASSO, least absolute shrinkage and selection operator.

Bold with red cells = best-performing algorithm per metric and outcome.

For subjective happiness, Random Forest achieved the highest predictive accuracy (*R*^2^ = 0.154, RMSE = 0.869, MAE = 0.736), followed closely by Linear Regression (*R*^2^ = 0.153, RMSE = 0.869, MAE = 0.740) and LASSO (*R*^2^ = 0.153, RMSE = 0.869, MAE = 0.741). XGBoost and KNN produced intermediate results (*R*^2^ = 0.134 and 0.130, respectively). SVM yielded the weakest performance (*R*^2^ = 0.127, RMSE = 0.948), indicating that the radial basis function kernel did not confer predictive advantage over linear architectures for this outcome.

For life satisfaction (SWLS), the performance hierarchy is partially reversed. Linear Regression and LASSO achieved the highest accuracy (*R*^2^ = 0.129, RMSE = 1.317, MAE = 1.101), followed by Random Forest (*R*^2^ = 0.107, RMSE = 1.344). KNN (*R*^2^ = 0.105, RMSE = 1.356) and XGBoost (*R*^2^ = 0.107, RMSE = 1.368) produced comparable intermediate results. SVM delivered the lowest accuracy (*R*^2^ = 0.073, RMSE = 1.500), yielding the lowest *R*^2^ across all models and both outcomes combined. The near-equivalence of Linear Regression and LASSO across both outcomes (delta-*R*^2^ < 0.002 in each case) indicates negligible L1-induced coefficient shrinkage, indicating that all four predictors contributed non-redundant variance without requiring variable selection.

The slopegraph in Figure 2 shows that the largest *R*^2^ difference between outcomes occurred for SVM (delta = 0.054, favoring happiness), while Linear Regression and LASSO showed the smallest difference (delta < 0.002), suggesting that linear models generalize with comparable accuracy across both constructs. The waterfall chart in Figure 3 shows that, for SWLS, SVM had the highest cumulative error (RMSE = 1.500), while Linear Regression and LASSO had the lowest (RMSE = 1.317), a span of 0.183 RMSE units across the algorithm set. Despite this range, all models yielded modest overall *R*^2^ values (0.073–0.154), consistent with the multi-determined etiology of subjective wellbeing.

### 3.4 Variable distributions across physical activity levels

**Figure 4:**
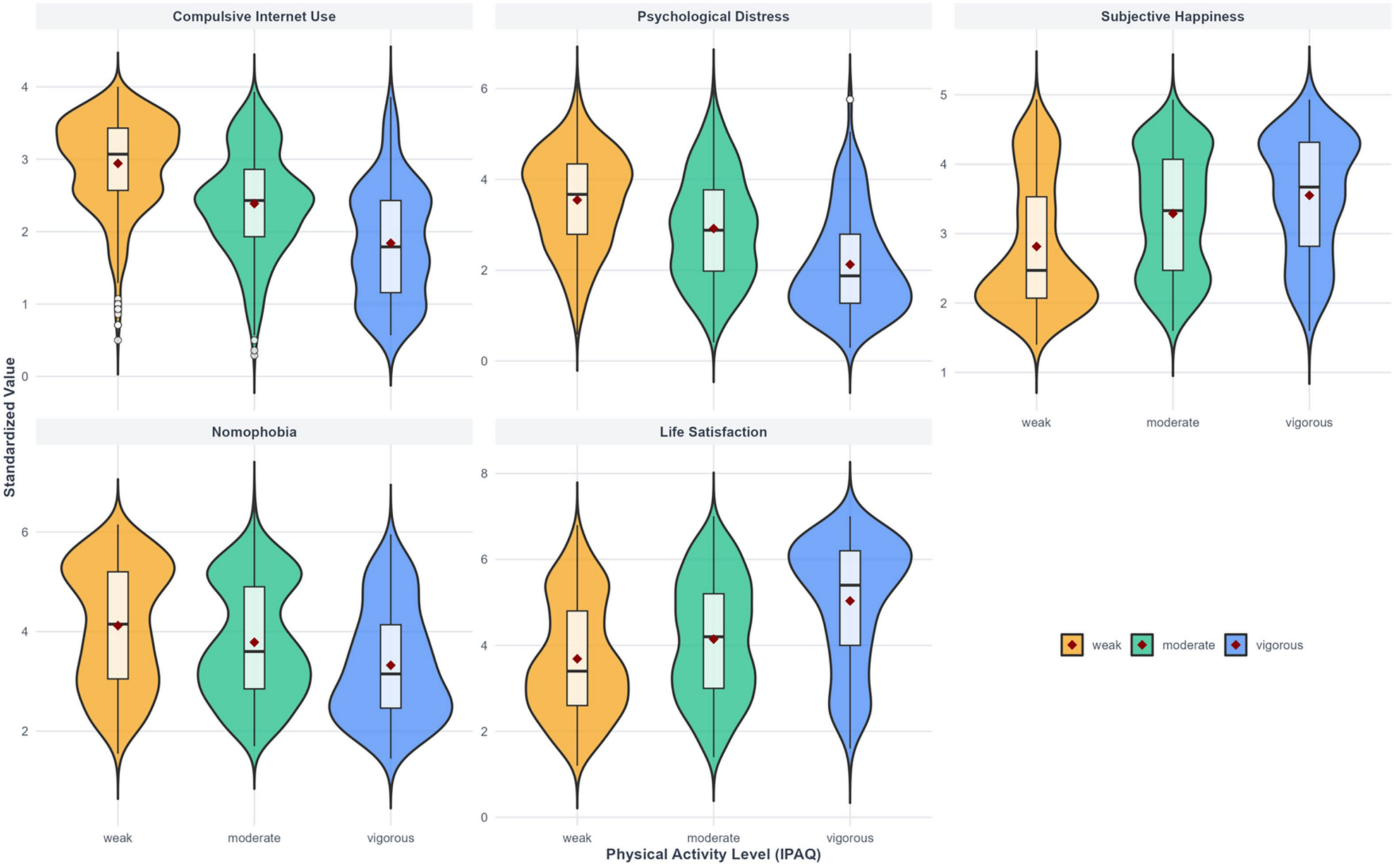
Distribution of all five study variables stratified by physical activity level (weak, moderate, vigorous) as classified by the IPAQ-SF. Each panel combines a violin plot (probability density) with an interquartile boxplot. Red points indicate group means; white boxes indicate interquartile ranges. CIUS, Compulsive Internet Use Scale; DASS-21, Depression Anxiety Stress Scales-21; SWLS, Satisfaction with Life Scale; IPAQ-SF, International Physical Activity Questionnaire Short Form.

Violin plots combined with interquartile boxplots, presented in Figure 4, display variable distributions stratified by IPAQ-classified physical activity level (weak, moderate, vigorous) across all five study constructs. A consistent, monotonic gradient was observed for each variable. For CIUS and DASS-21, the weak activity group exhibited the highest medians and widest distributional spread; the vigorous group showed the lowest medians and greatest distributional homogeneity, indicating greater psychological uniformity among physically active individuals. A parallel, attenuated gradient was observed for nomophobia, with substantial distributional overlap across the three levels. For SWLS and subjective happiness, the gradient was reversed: vigorous activity was associated with the highest medians and the most favorable distributional profiles, while weak activity was associated with the lowest wellbeing scores. The moderate activity group consistently occupied an intermediate position across all variables, supporting a monotonic dose–response relationship. Peak adverse scores (CIUS, DASS-21, nomophobia) were observed in the weak activity group, whereas peak positive scores (SWLS, happiness) were observed in the vigorous activity group. These findings indicate that physical activity level is systematically associated with both risk-related and wellbeing-related psychological outcomes, with incremental activity conferring graded protective effects.

## 4 Discussion

The present study examined the combined predictive utility of compulsive internet use, psychological distress, physical activity level, and nomophobia for life satisfaction and subjective happiness in a community-based Arabic-speaking sample, comparing six machine learning algorithms across both wellbeing outcomes. Four principal findings emerge. Psychological distress (DASS-21) exerted the strongest inverse associations with both outcomes (happiness: *r* = −0.40; SWLS: *r* = −0.37). Random Forest achieved superior predictive accuracy for happiness (*R*^2^ = 0.154, RMSE = 0.869), while Linear Regression and LASSO were optimal for life satisfaction (*R*^2^ = 0.129, RMSE = 1.317). Nomophobia showed negligible direct linear relationships with both wellbeing outcomes. Physical activity demonstrated a consistent, monotonic dose–response gradient across all five study constructs. These findings collectively advance understanding of how behavioral and psychological risk factors interact to shape subjective wellbeing in underrepresented Arabic-speaking populations.

The marginal but consistent superiority of Random Forest over linear models for subjective happiness, alongside the equivalence of Linear Regression and LASSO for life satisfaction, reflects a theoretically coherent pattern grounded in the tripartite model of subjective wellbeing (Diener, 1984). Subjective happiness is an affect-laden, momentarily responsive state whose determinants interact in configural, non-additive ways: for instance, the joint presence of high psychological distress and high compulsive internet use may suppress happiness beyond their individual additive effects. Random Forest captures such interaction structures without requiring explicit specification, which accounts for its marginal advantage (Fife and D'Onofrio, 2023). Life satisfaction, by contrast, is a stable cognitive-evaluative judgment that aggregates perceived circumstances across life domains (Diener, 1984); its determinants relate to outcomes through more monotonic, additive pathways that linear models characterize with comparable efficiency. This distinction is supported by the observation that the delta-*R*^2^ between Linear Regression and LASSO was less than 0.002 for both outcomes, confirming that all four predictors contributed non-redundant, largely additive variance to life satisfaction. The differential algorithm performance therefore reflects not a failure of linear models for happiness, but rather the greater configurality of affective wellbeing relative to its cognitive-evaluative counterpart. Kim et al. (2023) applied a random forest model to a nationally representative Korean sample (*N* = 44,320) to predict adolescent happiness, obtaining an *R*^2^ of 0.37 after including a broad range of psychosocial variables, and concluded that the algorithm’s capacity to capture psychosocial interactions accounted for its advantage over parametric alternatives. The considerably more modest *R*^2^ values observed in the present study (0.073–0.154) reflect the narrower predictor set employed and are consistent with a fundamental characteristic of wellbeing research: subjective wellbeing is substantially determined by genetic predispositions, personality traits, social relationships, and economic circumstances, variables that were necessarily outside the scope of the present cross-sectional design (Diener et al., 2018). The dominance of linear models for SWLS corroborates the theoretical distinction, formalized by Diener (1984), between life satisfaction as a stable cognitive-evaluative judgment and happiness as an affect-laden, momentarily responsive state.

The identification of DASS-21 as the strongest negative predictor across both outcomes is consistent with a robust cross-cultural literature. The negative association between psychological wellbeing and internet addiction (*r* = −0.417; *p* < 0.001) underscores the detrimental effects of compulsive online behaviors on mental health, while internet addiction has been found to disrupt social relationships, reduce real-world interactions, and generate feelings of loneliness and emotional instability. The slightly stronger association of DASS-21 with happiness than with life satisfaction (*r* = −0.40 vs. *r* = −0.37) aligns with the tripartite model of subjective wellbeing (Diener, 1984), in which negative affect is conceptualized as a direct hedonic component that fluctuates with current psychological state, whereas cognitive life evaluations are more buffered from transient distress. From an intervention standpoint, this distinction indicates that reductions in depression, anxiety, and stress should be expected to produce larger immediate gains in happiness than in life satisfaction, with the latter requiring more durable changes in perceived life circumstances. The *R*^2^ values observed across all models (0.073–0.154) are consistent with the established ceiling of individual-level subjective wellbeing prediction: even with large, representative samples and comprehensive predictor sets, ML models of wellbeing routinely yield *R*^2^ values below 0.15, reflecting the substantial contribution of genetic predispositions, stable personality traits, and unmeasured social and economic determinants (Oparina et al., 2025). The practical utility of the present models therefore resides in the reliable, cross-validated ranking of modifiable behavioral predictors within a four-predictor theoretically driven framework, rather than in aggregate predictive power. Within these constraints, the consistent identification of DASS-21 and CIUS as dominant predictors, and of physical activity as a monotonic protective gradient, provides actionable targets for population-level screening and intervention. The strong positive association between CIUS and DASS-21 (*r* = 0.48), the highest bivariate correlation in the matrix, likely reflects bidirectionality: distressed individuals may use the internet compulsively as a mood-regulation strategy, while excessive use exacerbates symptoms through disrupted sleep and social displacement (Ortuño-Sierra et al., 2022).

The near-zero bivariate correlations between nomophobia and both SWLS (*r* = −0.02) and happiness (*r* = 0.11) challenge the assumption that fear of mobile phone unavailability constitutes an independent risk factor for reduced wellbeing. This finding aligns with a recent cross-sectional study among adults (*N* = 306) that reported no significant association between nomophobia and life satisfaction, while documenting significant associations with phubbing behavior and time spent online (Ferreira et al., 2025). A systematic review and meta-analysis established that anxiety (*r* = 0.31, 95% CI: 0.25–0.38), smartphone addiction (*r* = 0.39, 95% CI: 0.04–0.75), and insomnia (*r* = 0.56, 95% CI: 0.38–0.75) are positively associated with nomophobia, indicating that nomophobia’s psychological consequences are channeled principally through anxiety and compulsive use constructs rather than through a direct effect on global wellbeing. The near-zero correlation in the present study likely reflects full mediation of the nomophobia-wellbeing pathway through DASS-21 and CIUS. Loneliness is positively associated with nomophobia, with smartphone attachment mediating this relationship, suggesting additional indirect pathways that remain to be tested in Arabic-speaking populations through formal mediation analysis.

The consistent monotonic gradient across all five constructs as a function of IPAQ-classified physical activity level constitutes one of the most robust findings of the present study. Vigorous activity was associated with the lowest CIUS, DASS-21, and nomophobia scores, as well as the highest SWLS and happiness scores; weak activity corresponded uniformly to the most adverse profiles. Positive outcome measures, including life satisfaction and positive affect as core components of mental wellbeing, show robust associations with physical activity across multiple domains, while negative outcomes, including psychological distress, stress, and negative affect, show consistent inverse associations. Mahindru et al. (2023) further demonstrated that regular exercise enhances mood and self-esteem while reducing tendencies toward stress, with neurobiological mechanisms including endorphin release, modulation of the HPA axis, and neuroplasticity accounting for the mental health benefits of sustained activity. The finding that moderate activity was associated with meaningfully better outcomes than weak activity across all constructs carries particular clinical relevance: it indicates that the protective threshold is accessible without requiring vigorous exertion, lowering the behavioral barrier for population-level interventions. Reductions in problematic mobile phone use significantly mediate improvements in depression and sleep disorders following mindfulness-based interventions, suggesting that physical activity programs that simultaneously displace sedentary digital behaviors may confer compounded benefits through both direct neurobiological and indirect behavioral pathways.

### 4.1 Limitations

Before turning to limitations, it is appropriate to acknowledge that alternative analytical approaches could have been applied to the present dataset. Structural equation modeling (SEM) would permit simultaneous estimation of latent variable relationships and formal testing of mediation pathways; however, SEM requires model specification *a priori* and cannot accommodate the non-linear interactions that the present ML framework was designed to detect. Regularized regression approaches such as ridge or elastic net extend beyond LASSO while remaining parametric; these were implicitly represented by LASSO in the present comparison. Bayesian hierarchical modeling would provide credible interval estimation and coherent uncertainty propagation but requires informative priors and substantially greater computational resources. The ML pipeline adopted here was selected because it (a) makes minimal parametric assumptions, (b) permits direct comparison of algorithms spanning both linear and non-linear families on held-out data, and (c) generates variable importance metrics without requiring path specification.

The cross-sectional design precludes causal inference; the directionality of associations between physical activity, compulsive internet use, and wellbeing cannot be established from the present data. All measures relied on self-report. Physical activity assessment via the IPAQ-SF is particularly susceptible to recall bias and social desirability effects, which may underestimate sedentary behavior and inflate moderate-to-vigorous activity classification. Device-based assessment using wrist-worn accelerometers or multi-sensor wearables provides continuous, epoch-level intensity data that are free from these biases and would substantially improve both measurement precision and the granularity of dose–response characterization in future work. The modest *R*^2^ values indicate meaningful omitted variance attributable to personality traits, sleep quality, social support, and economic status. Recruitment through convenience and snowball sampling limits generalizability to the broader Tunisian and Arab world population. Mediation and moderation pathways, particularly the hypothesized indirect transmission of nomophobia effects through DASS-21 and CIUS, remain untested. The hypothesized indirect transmission of nomophobia effects through DASS-21 and CIUS remains untested, as the cross-sectional design does not permit formal mediation or moderation analysis. Structural equation modeling or longitudinal path analysis in future studies should explicitly test whether psychological distress and compulsive internet use fully mediate the nomophobia–wellbeing relationship, and whether physical activity moderates these pathways.

### 4.2 Practical recommendations

Physical activity promotion should be prioritized as a scalable, low-cost public health intervention in Arabic-speaking community settings. Based on the observed dose–response relationship, even moderate-intensity activity (e.g., ≥150 min per week) may confer meaningful psychological benefits. Community-level initiatives, including school-based physical education programs and workplace wellness interventions, should incorporate structured physical activity components to reduce psychological distress and improve overall wellbeing. Routine mental health screening should be integrated into educational and occupational settings using brief, validated tools such as the Depression Anxiety Stress Scales (DASS-21). Individuals presenting with moderate-to-high distress scores should be prioritized for early intervention, as psychological distress emerged as the strongest modifiable predictor of both life satisfaction and subjective happiness. Digital literacy and behavioral self-regulation programs should be implemented to address maladaptive internet use. These programs may include time-management strategies, awareness of problematic usage patterns, and cognitive-behavioral techniques to reduce compulsive engagement. Given the observed association between compulsive internet use and psychological distress (*r* = 0.48), interventions targeting digital behavioral risks should be integrated within broader mental health promotion frameworks. Finally, clinicians and practitioners should avoid focusing solely on nomophobia as a treatment target. The findings suggest that its impact on wellbeing is likely indirect and mediated through psychological distress and compulsive internet use. Therefore, intervention strategies should prioritize underlying psychological mechanisms rather than symptom-specific manifestations.

## 5 Conclusion

The present study demonstrates that psychological distress emerged as the strongest negative predictor of life satisfaction and subjective happiness in an Arabic-speaking community sample, whereas compulsive internet use showed moderate but consistent associations with reduced wellbeing. In contrast, physical activity exhibited a clear dose–response relationship across all psychological constructs, reinforcing its role as a transdiagnostic protective health behavior. Different machine learning models showed outcome-specific performance, with tree-based approaches performing slightly better for subjective happiness and linear models for life satisfaction. However, the findings primarily emphasize the importance of identifying key behavioral and psychological determinants rather than the superiority of any single predictive approach. Notably, nomophobia showed negligible direct associations with wellbeing outcomes, suggesting that its effects may be indirect and potentially mediated through psychological distress and compulsive internet use. From a health psychology perspective, these results highlight the importance of targeting psychological distress and maladaptive digital behaviors while promoting physically active lifestyles as part of integrated mental health strategies. Future research should adopt longitudinal designs to clarify causal pathways, incorporate broader psychosocial and personality-related variables, and examine mechanisms of mediation linking digital behavioral risks to wellbeing. Such approaches will support the development of more targeted and effective interventions to improve population mental health. Overall, the findings support a multidimensional behavioral framework in which psychological distress and digital behavioral risks represent key intervention targets for improving subjective wellbeing.

## Data Availability

The raw data supporting the conclusions of this article will be made available by the authors, without undue reservation.
